# Confluence Stones at the Junction of the Cystic Duct and an Aberrant Anterior Sectoral Bile Duct: A Case Report

**DOI:** 10.70352/scrj.cr.26-0430

**Published:** 2026-10-17

**Authors:** Takeshi Kawai, Yuichiro Kato, Naoya Yamaguchi, Fumitoshi Mizutani, Jiro Kawakami, Naoya Torii, Tomohiko Murakami, Ryoichi Shimizu, Hirotaka Ito

**Affiliations:** Department of Surgery, Nagoya Ekisaikai Hospital, Nagoya, Aichi, Japan

**Keywords:** confluence stone, aberrant anterior sectoral bile duct, biliary anomaly, communicating accessory bile duct

## Abstract

**INTRODUCTION:**

Confluence stones usually arise when gallstones impacted in the cystic duct migrate toward the biliary confluence. Surgical treatment can be technically demanding because of chronic inflammation and distorted anatomy. Confluence stones at the junction between the cystic duct and an aberrant bile duct are extremely rare. We report a successfully treated case of confluence stones arising at the junction of the cystic duct and an aberrant anterior sectoral bile duct with a communicating accessory bile duct.

**CASE PRESENTATION:**

A 38-year-old woman presented with abdominal pain, and biliary stones were detected. Magnetic resonance cholangiopancreatography demonstrated an aberrant anterior sectoral bile duct draining independently into the common bile duct. Biliary stones were identified both within this duct and at its junction with the cystic duct. Endoscopic retrograde cholangiography confirmed the biliary anatomy and additionally revealed a communicating accessory bile duct between the anterior and posterior sectoral ducts. The stones within the distal portion of the aberrant duct were removed endoscopically, but the impacted confluence stones could not be extracted. Therefore, open surgery was performed. After exposure of the junction, the confluence stones were removed through a semicircumferential incision. Complete stone clearance was confirmed by intraoperative cholangioscopy and cholangiography. The patient was discharged on POD 11 with no bile leakage. At 14 months postoperatively, she remained asymptomatic and had experienced no episodes of cholangitis.

**CONCLUSIONS:**

We report a rare case of confluence stones arising at the junction of the cystic duct and an aberrant anterior sectoral bile duct. Preoperative assessment of the drainage territory and intraoperative evaluation of the duct diameter, ischemia, and tissue fragility are essential for appropriate and safe repair of the bile duct defect. The repair strategy should not rely on presumed bypass flow through a communicating accessory bile duct because its functional capacity cannot be reliably predicted.

## Abbreviations

DIC-CT
: drip infusion cholangiography with CT
ERC
: endoscopic retrograde cholangiography
MRCP
: magnetic resonance cholangiopancreatography

## INTRODUCTION

Confluence stones are generally considered to result from gallstone impaction at the cystic duct, followed by gradual migration toward the biliary confluence.^1)^ First, chronic inflammation around the biliary confluence may obscure the local anatomy and make surgery technically demanding,^2)^ with a potential risk of bile duct injury or postoperative biliary stricture.

Biliary anatomical variations, such as aberrant sectoral bile ducts and communicating accessory bile ducts, are clinically important because they increase the risk of biliary injury.^3)^ However, the occurrence of confluence stones at the junction between the cystic duct and an aberrant bile duct is extremely rare. We herein report a rare case of confluence stones arising at the junction of the cystic duct and an aberrant anterior sectoral bile duct with a communicating accessory bile duct, successfully treated by stone extraction, cholecystectomy, and primary closure of the bile duct defect.

## CASE PRESENTATION

A 38-year-old woman presented to our emergency department with abdominal pain 4 months before referral to our department. Her medical history included asthma and cervical dysplasia. CT revealed biliary stones extending from the gallbladder neck to the cystic duct (**Fig. 1**). Laboratory findings showed no evidence of liver dysfunction or inflammation. MRCP demonstrated that the anterior sectoral bile duct drained independently into the common bile duct (**Fig. 2**). Filling defects were observed at the junction between the cystic duct and this aberrant anterior sectoral duct, consistent with confluence stones, and additional stones were present within the aberrant duct itself. ERC confirmed the same biliary anatomy. In addition, a communicating accessory bile duct between the anterior and posterior sectoral ducts was demonstrated by guidewire passage (**Fig. 3**). Neither MRCP nor ERC showed dilation of the intrahepatic anterior sectoral duct despite localized dilation at the stone-impacted junction. These findings suggested that the communicating accessory bile duct may have provided an alternative drainage pathway for the anterior sector. The stones within the distal portion of the anterior sectoral duct were removed endoscopically, but a cluster of confluence stones remained impacted at the junction and could not be extracted. Therefore, surgical removal of the remaining stones was performed. A preoperative schematic diagram of the biliary confluence is shown in **Fig. 4**.

**Fig. 1:**
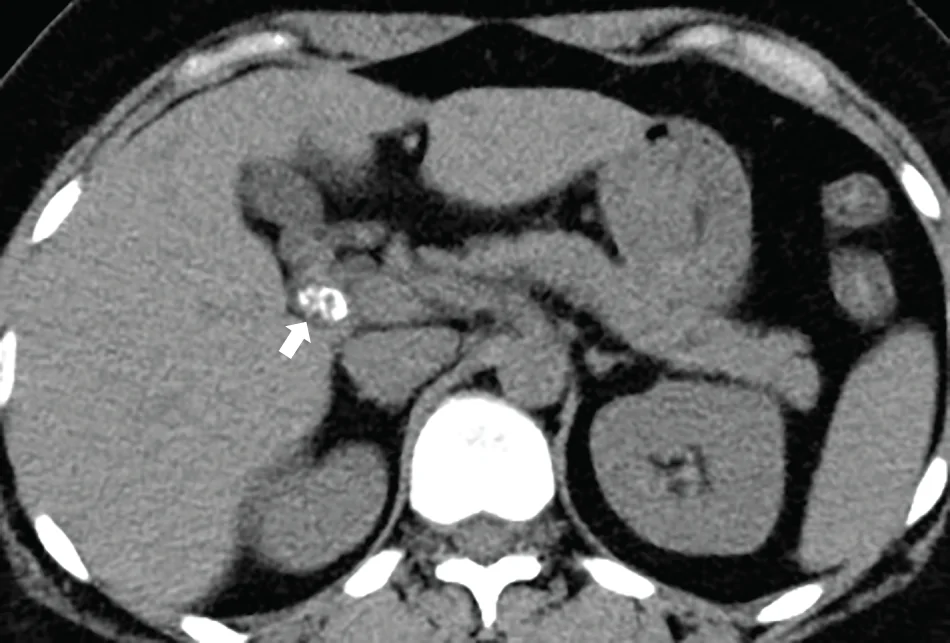
CT findings. Biliary stones extending from the gallbladder neck to the cystic duct are shown (arrow).

**Fig. 2:**
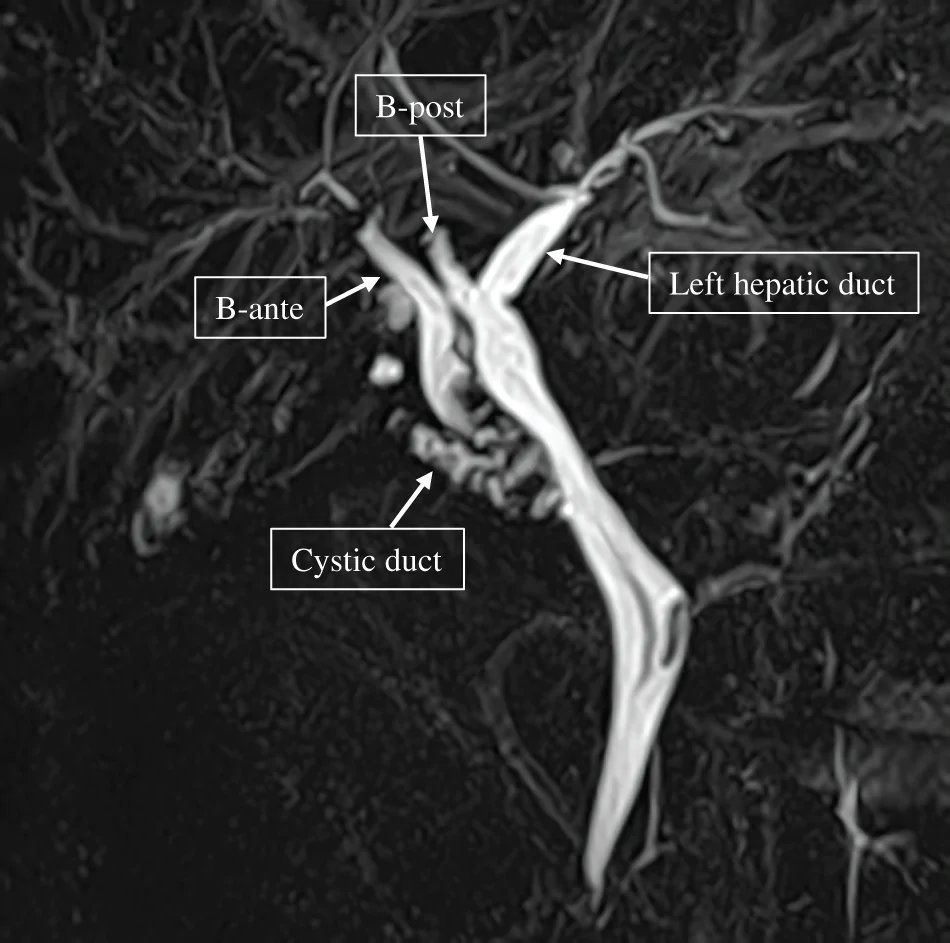
MRCP findings. An aberrant B-ante is demonstrated draining independently into the common bile duct. Filling defects are observed at the junction of the cystic duct and the aberrant B-ante, as well as within the distal portion of the aberrant duct. Despite localized dilation at the stone-impacted junction, no dilation of the intrahepatic anterior sectoral duct is observed. B-ante, anterior sectoral bile duct; B-post, posterior sectoral bile duct; MRCP, magnetic resonance cholangiopancreatography

**Fig. 3:**
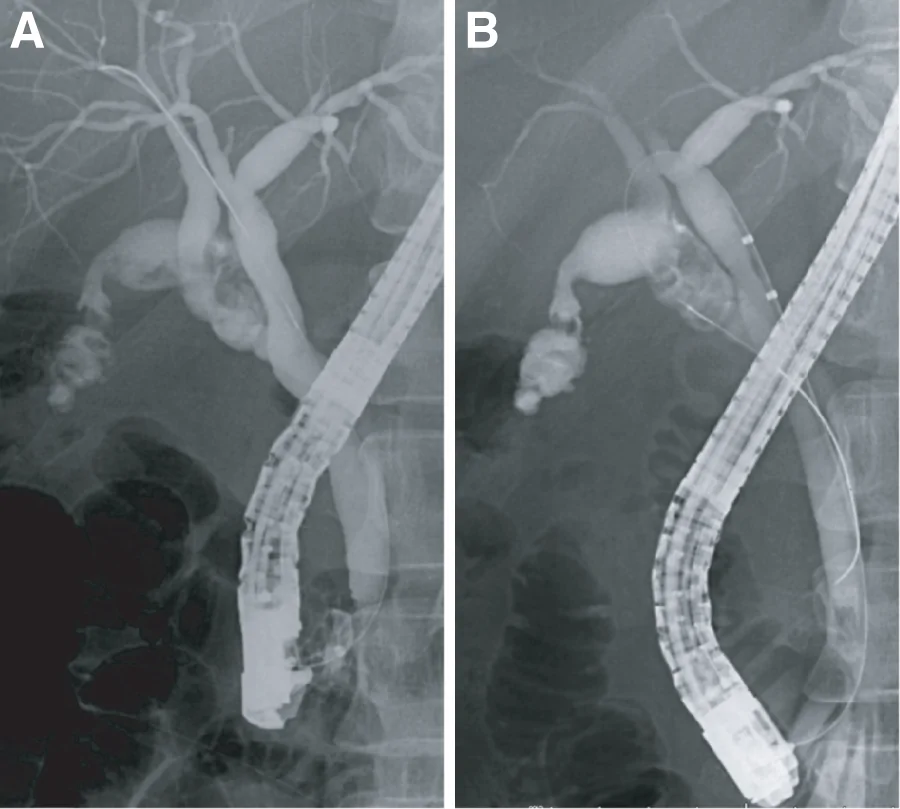
ERC findings after endoscopic removal of stones from the distal portion of the anterior sectoral bile duct. (**A**) A guidewire was advanced from the posterior sectoral duct through the communicating accessory bile duct into the aberrant anterior sectoral bile duct. (**B**) The guidewire was then passed retrogradely through the aberrant anterior sectoral bile duct into the common bile duct. A cluster of confluence stones remained impacted at the junction and could not be extracted endoscopically. ERC, endoscopic retrograde cholangiography

**Fig. 4:**
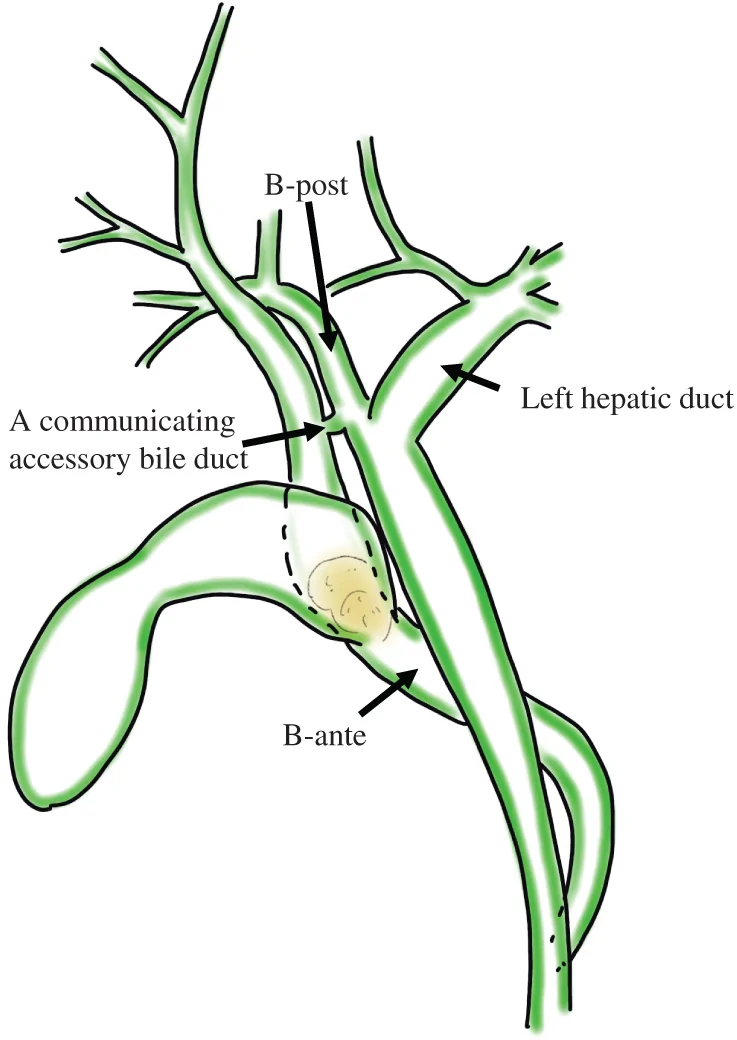
Preoperative schematic diagram of the biliary anatomy. The aberrant B-ante drains independently into the common bile duct and communicates with the posterior sectoral duct via a communicating accessory bile duct. Confluence stones are present at the junction of the cystic duct and the aberrant B-ante. B-ante, anterior sectoral bile duct; B-post, posterior sectoral bile duct

Open surgery via an upper midline laparotomy was performed. After the gallbladder was detached from the liver bed, the junction of the cystic duct and the aberrant anterior sectoral bile duct was exposed. The confluence was dilated by the impacted stones. To facilitate access to the impacted confluence stones, a semicircumferential incision was made directly at the confluence without leaving a cystic duct stump. The cluster of impacted stones at the confluence was removed manually. Complete stone clearance from the aberrant anterior sectoral bile duct was confirmed by intraoperative cholangioscopy and cholangiography. For cholangiography, contrast medium was injected through a 6-Fr catheter inserted via the bile duct incision. Because the aberrant duct had a sufficient diameter and showed no damage or ischemia, and the junction had been dilated by the impacted stones, the risk of postoperative biliary stricture was considered low. Therefore, the incision at the confluence was extended circumferentially to complete cholecystectomy without preserving a gallbladder cuff for choledochoplasty, and the bile duct defect was repaired by primary closure with 4-0 monofilament absorbable sutures. Finally, a 6.5-mm multichannel drain was placed in the foramen of Winslow (**Fig. 5**).

**Fig. 5:**
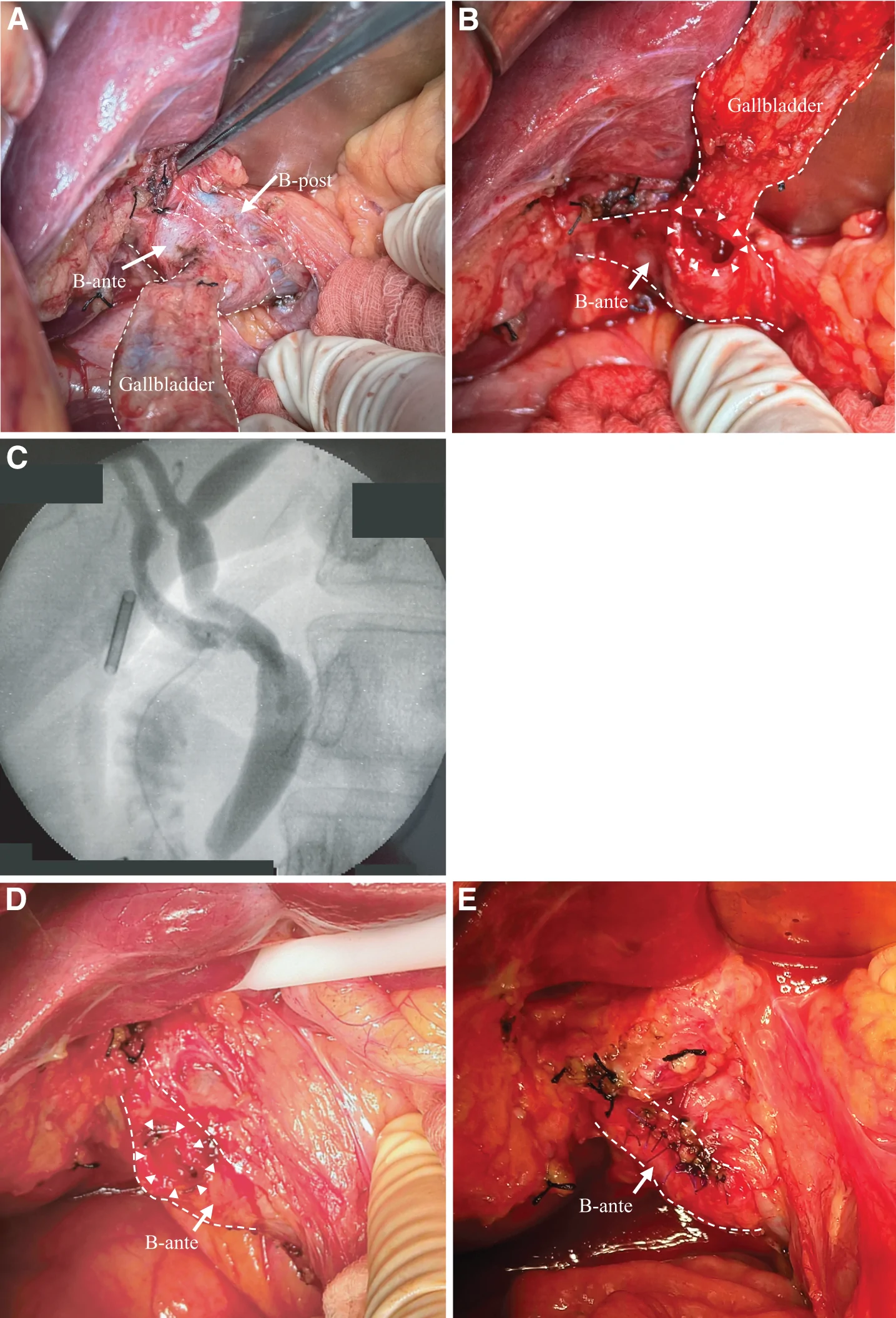
Intraoperative findings. (**A**) The junction between the cystic duct and the aberrant B-ante was exposed. (**B**) A semicircumferential incision was made directly at the junction (arrowheads), leaving no cystic duct stump, and the impacted stones were removed. (**C**) Intraoperative cholangiography through a 6-Fr catheter inserted via the bile duct incision confirmed the expected biliary anatomy and complete stone clearance. (**D**) The incision at the junction was extended circumferentially to complete cholecystectomy. The bile duct defect (arrowheads) was located in the aberrant B-ante. (**E**) The bile duct defect was repaired by primary closure with 4-0 monofilament absorbable sutures. B-ante, anterior sectoral bile duct; B-post, posterior sectoral bile duct

The postoperative course was complicated by an intra-abdominal abscess. CT performed on POD 3 revealed a localized fluid collection just below the abdominal wall in the right upper quadrant, separate from the bile duct repair site. The surgical drain placed in the foramen of Winslow yielded serous output with a normal bilirubin concentration, indicating no evidence of bile leakage. The fluid collection was therefore diagnosed as a postoperative intra-abdominal abscess, presumably related to intraoperative contamination, and antimicrobial therapy with piperacillin–tazobactam was initiated on the same day. On POD 7, the fluid collection was aspirated under US guidance, and the bilirubin concentration in the aspirated fluid was not elevated, providing further evidence against bile leakage. The surgical drain was subsequently removed. On POD 10, the inflammatory markers had normalized, and antimicrobial therapy was discontinued. She was discharged on POD 11. Pathological evaluation identified chronic cholecystitis with no evidence of malignancy. At 14 months postoperatively, the patient remained asymptomatic and had experienced no episodes of cholangitis. Follow-up MRCP was not performed because the patient was unable to cooperate with the examination.

## DISCUSSION

Confluence stones are generally thought to result from gallstone impaction at the cystic duct with gradual migration toward the biliary confluence.^1)^ Surgery is the primary treatment for confluence stones because of the need for removal of the gallbladder.^4)^ However, surgical treatment can be technically difficult because chronic inflammation around the confluence leads to dense adhesions and distortion of the biliary anatomy.^2)^ Furthermore, careful management is required for repair of the biliary tract defect because of the risk of postoperative bile leakage and biliary stricture.^5)^

In the present case, the most notable anatomical feature was an aberrant anterior sectoral bile duct draining independently into the common bile duct. Aberrant bile ducts are clinically important because they increase the risk of misidentification and biliary injury.^3)^ Previous reports have shown that anomalous ducts most commonly involve the posterior sector, whereas involvement of the anterior sectoral duct is less frequent.^6,7)^ In addition, the coexistence of a communicating accessory bile duct further complicated the biliary anatomy in our patient. Goor and Ebert defined communicating accessory bile ducts as those that communicate between major bile ducts but do not drain any part of the liver.^8)^ Although cholecystectomy in patients with an aberrant bile duct or a communicating accessory bile duct has been reported,^9,10)^ we found no reported cases of confluence stones at the junction of the cystic duct and an aberrant bile duct. The present case is considered extremely rare because confluence stones developed at the junction between the cystic duct and an aberrant bile duct draining the anterior sector.

Another important point in this case was the method of repair after stone extraction and cholecystectomy. Reported surgical options for bile duct defects associated with confluence stones or bile duct injury include primary closure, choledochoplasty, T-tube drainage, and biliary-enteric reconstruction.^4,5,11–14)^ To avoid postoperative complications, management of bile duct defects should be individualized according to the size of the defect, duct diameter, and extent of inflammation or ischemia.^14,15)^ Small defects may be managed by primary closure, and choledochoplasty using a gallbladder wall patch to restore duct caliber can be considered when the defect is larger and primary closure is likely to narrow the lumen.^11,12)^ T-tube drainage may be a useful option in cases in which a gallbladder patch cannot be created, or the quality of tissue repair is uncertain, because it serves to decompress the bile duct and helps to shape the duct wall.^4)^ In contrast, extensive defects or severely inflamed duct tissue generally require biliary-enteric reconstruction, such as Roux-en-Y hepaticojejunostomy.^5)^ This strategy is consistent with the 2020 World Society of Emergency Surgery guidelines for the detection and management of bile duct injury during cholecystectomy, which state that direct repair with or without T-tube placement may be considered for minor bile duct injuries, whereas Roux-en-Y hepaticojejunostomy is recommended for major injuries associated with tissue loss or ischemia.^16)^ In particular, in cases of confluence stones at the junction between the cystic duct and an aberrant bile duct, careful intraoperative management is required because the defect in the aberrant duct is relatively large, given that the aberrant duct is narrower than the common bile duct. Because the diameter of an aberrant bile duct has been reported to be proportional to the area it drains,^17)^ detailed preoperative evaluation of its confluence pattern and drainage area using MRCP, DIC-CT, and ERC is considered important for appropriate repair of the bile duct defect. In the present case, the aberrant bile duct draining the entire anterior sector had a sufficient diameter, and the duct wall at the junction was in good condition and had already been dilated by the impacted stones. We therefore considered primary closure to be an appropriate and less invasive option for repair. The absence of postoperative bile leakage and the favorable clinical course without cholangitis during 14 months of follow-up supported the feasibility of this strategy. However, asymptomatic biliary stricture cannot be excluded because follow-up MRCP was not obtained.

An important limitation in interpreting the successful primary closure in this case is that the communicating accessory bile duct may have functioned as a bypass route for bile flow. This possibility is consistent with preoperative MRCP and ERC findings, which showed no dilation of the intrahepatic anterior sectoral duct despite localized dilation at the stone-impacted junction. The bypass flow may have reduced the intraductal pressure at the repair site, thereby creating favorable conditions for primary closure of the bile duct defect. However, to our knowledge, bile flow through a communicating accessory bile duct and its effect on intraductal pressure have not been quantitatively evaluated. Therefore, it is difficult to predict preoperatively whether the communicating accessory bile duct would function as an effective postoperative bypass route. In the challenging setting of confluence stones at the junction between an aberrant bile duct and the cystic duct, a safe method for repairing the bile duct defect should be selected without assuming adequate bypass flow, even if a communicating accessory bile duct is present. In the present case, primary closure was selected rather than choledochoplasty because the defect could be closed without luminal narrowing and the vascularity of the gallbladder cuff cannot always be guaranteed.^4)^ However, choledochoplasty, T-tube drainage, or biliary-enteric reconstruction should be considered when the aberrant bile duct has a small caliber, the defect is large, or the tissue quality is poor.

In summary, we report a rare case of confluence stones arising at the junction of the cystic duct and an aberrant anterior sectoral bile duct, accompanied by a communicating accessory bile duct. Our case adds useful practical information regarding operative decision-making in this technically challenging setting. When confluence stones develop in association with an aberrant bile duct, detailed preoperative assessment of the confluence pattern, drainage territory, and intraoperative evaluation of bile duct diameter and the presence of ischemia or fragility are essential for selecting an appropriate method of repair.

## CONCLUSIONS

We report a rare case of confluence stones arising at the junction of the cystic duct and an aberrant anterior sectoral bile duct. Detailed preoperative and intraoperative evaluation of the aberrant bile duct is essential for safe surgical management and appropriate repair of the bile duct defect.

## DECLARATIONS

### Use of artificial intelligence (AI) tool

The authors acknowledge the use of ChatGPT, developed by OpenAI, for English-language editing. The authors take full responsibility for the content of this manuscript.

### Availability of data and materials

All relevant data supporting the findings of this case report are available upon request.
